# Fuzzy C-Means Clustering and Energy Efficient Cluster Head Selection for Cooperative Sensor Network

**DOI:** 10.3390/s16091459

**Published:** 2016-09-09

**Authors:** Dost Muhammad Saqib Bhatti, Nasir Saeed, Haewoon Nam

**Affiliations:** 1 Department of Electronics and Communication Engineering, Hanyang University, Ansan 15588, Korea; saqib@hanyang.ac.kr; 2 Faculty of Computer Science, Iqra National University, Peshawar, Pakistan; mr.nasir.saeed@ieee.org

**Keywords:** sensor networks, energy efficiency, clustering

## Abstract

We propose a novel cluster based cooperative spectrum sensing algorithm to save the wastage of energy, in which clusters are formed using fuzzy c-means (FCM) clustering and a cluster head (CH) is selected based on a sensor’s location within each cluster, its location with respect to fusion center (FC), its signal-to-noise ratio (SNR) and its residual energy. The sensing information of a single sensor is not reliable enough due to shadowing and fading. To overcome these issues, cooperative spectrum sensing schemes were proposed to take advantage of spatial diversity. For cooperative spectrum sensing, all sensors sense the spectrum and report the sensed energy to FC for the final decision. However, it increases the energy consumption of the network when a large number of sensors need to cooperate; in addition to that, the efficiency of the network is also reduced. The proposed algorithm makes the cluster and selects the CHs such that very little amount of network energy is consumed and the highest efficiency of the network is achieved. Using the proposed algorithm maximum probability of detection under an imperfect channel is accomplished with minimum energy consumption as compared to conventional clustering schemes.

## 1. Introduction

Due to explosive demand for wireless communication during the last decade, broader spectrum resources are needed. However, spectrum resources are limited and are allocated according to a fixed spectrum assignment policy. The concept of sensing the spectrum was first presented by Mitola [1] to solve the problem of spectrum scarcity pointed by the Federal Communication Commission report [2]. The goal is to sense frequency band and utilize that band, if the licensed user called a primary user (PU) is not using it. Thus, the detection performance in the spectrum sensing is crucial to the performance of both PUs and the sensor network. The detection performance can be primarily determined on the basis of two metrics: probability of false alarm, which denotes the probability of a sensor declaring that a PU is present when the spectrum is actually free, and the probability of detection, which denotes the probability of a sensor declaring that a PU is present, given that the spectrum is indeed occupied by the PU. Since the detection avoids the interference with the PU and a false alarm reduces the spectral efficiency, it is usually required for optimal detection performance that the probability of detection is maximized subject to the constraint of the probability of false alarm.

There are several spectrum sensing techniques available in literature, some of which are matched filter detection [3], energy detection [4], cyclostationary detection [5], wavelet detection [6], and covariance detection [7]. Among them, energy detection is widely applied for sensing the spectrum as it does not require any prior knowledge of primary signals and has much lower complexity than the others [8,9,10,11,12,13]. Therefore, this paper also considers energy based detection.

The sensing decision of a single sensor may not be reliable enough due to shadowing, multipath fading and the time varying nature of wireless channels between sensors and PUs. To overcome these effects, the literature is brimming with cooperative spectrum sensing schemes to take advantage of spatial diversity [14,15,16,17]. In cooperative spectrum sensing, sensors of whole network share sensing information to the Fusion center (FC) which is combined to make the final decision. This can result in an excessive consumption of the network energy when large number of sensors are cooperating, therefore sensors are sometimes divided into clusters, which is called a cluster based cooperative spectrum sensing.

Each sensor delivers sensing information to the FC in one of the two ways: hard information, and soft information. In [15,16], hard information is considered, where each sensor sends only one bit of information regarding the detection of PUs. In soft information, accurate average energy values observed by all sensors are reported to FC, which are combined to make a final decision. It is shown in [18] that soft information combining outperforms hard combining. Hence, a soft combination is considered in this paper. Note that each sensor performs the spectrum sensing via sensing channel and forwards the sensing information to the FC via reporting channel.

Since each sensor consumes energy in sensing PU signals and reports that sensed energy to FC; consequently, cooperative spectrum sensing can cause high overall energy consumption in the whole network when a large number of sensors are involved in cooperation. To combat this problem, grouping the cooperative sensors into clusters [19,20,21] for cooperative sensing is an effective method to reduce the energy consumption.

The clustering techniques with respect to sensing efficiency have widely been shown in literature [19,20], but very few energy efficient schemes have been proposed [22,23,24,25,26], although the energy efficiency is one of the most important factors for the designing of sensor networks. To the best of the author’s knowledge, clustering schemes have mostly used perfect channels and have considered hard decisions. Thus, using imperfect channels, we have proposed a novel cluster based soft combining scheme for energy efficient spectrum sensing in sensor networks, in which maximum sensing efficiency is achieved with less consumption of energy. In [19], the authors have proposed a clustering method for spectrum sensing, in which sensors with largest reporting channel gain are selected as cluster heads (CHs) to reduce the reporting error. In [20], four clustering methods are shown to reduce the overhead depending on the location information of sensors. In [21], a cluster-based cooperative spectrum sensing scheme is proposed to address the control channel and sensing delay problems. However, these cluster-based spectrum sensing approaches do not focus on the energy consumption. In [22], authors have focused on energy consumption in cooperative spectrum sensing and have proposed a multi-hop cluster based cooperative spectrum sensing for reducing wastage of energy, in which CHs are selected based on distance between FC and sensors, reporting channel gain and energy level. However, based on these parameters, FC most likely select the nearest sensors to it. In [23], authors have proposed the method that uses the fuzzy c-means (FCM) technique for the cluster formation. However, there are few drawbacks, i.e., (i) each sensor forwards its sensing measurement to FC even though authors have divided the network into clusters; (ii) only making clusters does not always lead us to save energy, CH selection after making clusters plays a big role in the saving of energy; (iii) after cluster formation, each sensor forwards its sensing information to the FC for cluster decision, due to which a large amount of energy is consumed, and (iv) authors have considered the perfect channel that does not give assurance of the sensing efficiency. In [26], authors have also used FCM for the cluster formation and have selected the CH based on residual energy of the sensor. However, there are few flaws, such as: (i) authors have not shown energy efficiency of the network; (ii) the selection of CH based on only single parameter does not give certainty of energy efficiency; and (iii) the CH selection based on individual residual energy does not lead us to save energy, it only selects the CH based on amount of energy left in the sensor.

The above clustering schemes have not considered imperfect channels and have either focused on sensing efficiency or energy efficiency. By considering imperfect channels, we have proposed an algorithm that achieves maximum sensing efficiency and obtains the highest energy efficiency.

The main goal of this paper is to get the maximum probability of detection under an imperfect reporting channel, minimize the energy consumption by efficiently making the clusters and select CHs to improve the energy efficiency for the sensor networks. More specifically, a clustering based sensing scheme is proposed, in which clusters are made using the FCM technique to save energy consumption, and, furthermore, to save energy, CHs are selected based on four parameters: the location of sensors within cluster, location with respect to FC, sensor’s residual energy, and signal-to-noise ratio (SNR) of the reporting channel of the sensors. The main contributions of this paper are: firstly, the overall energy consumption of the whole networks is reduced using the proposed algorithm; secondly, maximum probability of detection is achieved under an imperfect reporting channel with smaller consumption of energy; and, finally, maximum efficiency of the network is achieved.

The rest of this paper is organized as follows. Section 2 describes the problem statement. Section 3 describes the system model. The proposed scheme is shown in Section 4. Simulation results are shown in Section 5. Finally, conclusions are summarized in Section 6.

## 2. Problem Statement

The detection performance of the network is determined on the basis of probability of false alarm ($P_{f}$) and probability of detection ($P_{d}$). The false alarm reduces the spectral efficiency of a sensor network and maximum probability of detection avoids the interference with PU. There are three phases in cooperative spectrum sensing; the first one is the sensing phase, in which all cooperative sensors perform local spectrum sensing; the second one is the reporting phase, in which local sensing data is reported to the FC in order to make the final decision by combining sensing measurement of all sensors, and the last one is the data transmission phase, in which data of sensors is transmitted if the PU is not active. Each sensor consumes energy on sensing the PU and reporting the sensed information to the FC due to the distance between them. Thus, as the number of sensors in the cooperative sensor network is increased, more energy is consumed.

**Figure 1:**
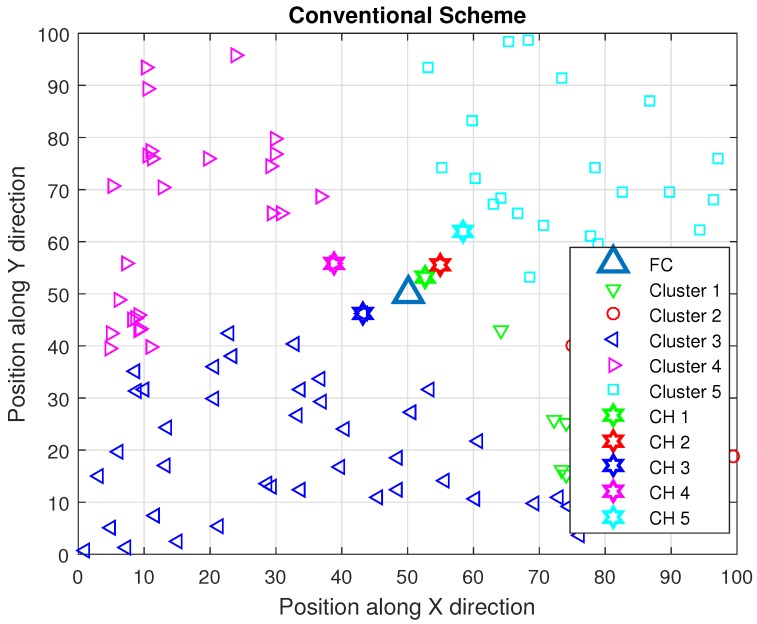
Conventional scheme.

To overcome the problem of energy consumption, many clustering methods are proposed, in which the whole network is divided into a small number of groups called clusters. Dividing the network into a small number of clusters saves energy, as FC has to receive the sensing information from CHs only, which is combined information of all cluster members. Every sensor forwards the sensing data to its CH and each CH combines the sensing information of its cluster member, which is forwarded to FC for the final decision. An energy efficient clustering method is dependent of two entities, one is the cluster formation and the other is the CH selection. Many of the authors have proposed cluster formation and CH selection methods, in which CHs are selected randomly or based on received signal strength indicator (RSSI) at FC from CHs, and, furthermore, CHs select their cluster members randomly or based on RSSI at CHs from sensors [24,25]. In [24], authors have proposed CH selection based on RSSI, energy level, reporting channel gain and distance between FC and CHs. In [22], authors have proposed a modified version of a well-known cluster making method low energy adaptive clustering hierarchy (LEACH), in which CHs are selected based on energy levels. However, all of these cluster formation methods have uneven distribution of clusters and most likely the nearest sensors to the FC are selected as CHs, which results in large distances between the CHs and their cluster members as shown in Figure 1 since all sensors forward their sensing measurement to their CHs . Hence, the large distance between the CHs and their cluster members causes huge energy consumption. In our proposed algorithm, the intention is to select the CH near its cluster members, but we also do not want it to be very far from FC. Thus, we have used FCM for the making of clusters and considered both distances, i.e., distance between CHs and their cluster members and distance between CHs and FC, for the selection of CH. The FCM is the technique that assigns degree of membership to a cluster for each sensor, which leads us to overcome the issue of uneven distribution. The FCM has been proved to enhance the network performance in terms of energy consumption [26].

The goal is to get the maximum probability of detection under an imperfect reporting channel with minimum energy consumption of the network by making energy efficient clusters and by selecting the best CH among each cluster. Our proposed algorithm makes the clusters using FCM and selects CHs based on sensor’s location, distance, residual energy and SNR. To be more specific, the idea is to divide the whole network into some number of clusters using FCM, and select the CHs based on the four parameters, location of sensors within cluster, location of sensors with respect to FC, SNR of the sensor’s reporting channel and sensor’s residual energy. Our proposed algorithm achieves its highest efficiency as the use of FCM reduces the energy consumption of the network, and selection of CHs based on our algorithm not only saves more energy but also achieves the highest performance of the network.

## 3. System Model

**Figure 2:**
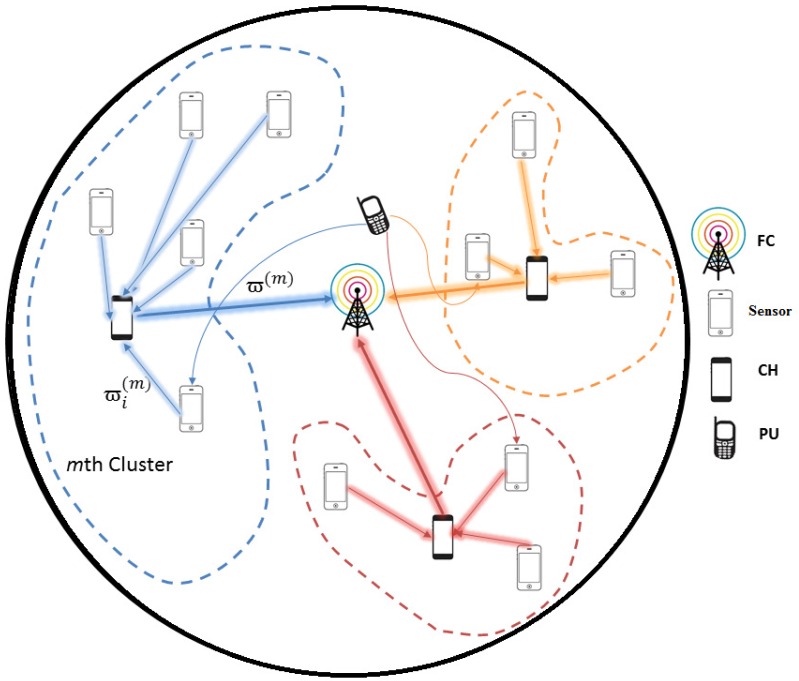
System model.

Consider a network of *N* cooperative sensors with one FC, which is divided into *M* number of clusters each with one CH and a PU. The sensors are randomly distributed as shown in Figure 2.

The topological structure of the sensor network is represented by a network graph *G*, with each vertex representing the position of sensor in two-dimensional space, i.e., $\theta_{i}={\left[x_{i},y_{i}\right]}^{T}$ for the *i*th sensor. We put an edge for the link between the *i*th and *j*th sensor if they are located within a certain communication range *r*; otherwise, there is no edge if they are out of communication. An edge is also defined for the link from a PU to each sensor if the PU is detected by the sensor through spectrum sensing. The objective of the sensor is to sense the spectrum and opportunistically utilize it if not used by a PU. Each cluster consists of a CH, which combines the sensing measurements from all sensors of that cluster and forwards that combined sensing measurement to the FC. Thus, the process of cooperative sensing starts with spectrum sensing performed individually at each sensor called local spectrum sensing. The time frame of cooperative spectrum sensing is divided into four sub frames, the sensing time $\left(T_{s}\right)$ of sensors, reporting time $\left(T_{c}\right)$ of cluster members to their CHs, reporting time $\left(T_{r}\right)$ of CHs to the FC and data transmission time $\left(T_{t}\right)$. Therefore, the total frame duration can be written as

(1) $$T=T_{s}+N_{m}T_{c}+MT_{r}+T_{t},$$

where $N_{m}$ is the number of sensors in the *m*th cluster, which is the largest cluster of the network in terms of sensors and *M* is the total number of clusters in the network. The performance of cooperative spectrum sensing can be analyzed by $P_{d}$, $P_{f}$, throughput, energy consumption and energy efficiency. The goal is to get the maximum detection performance under imperfect reporting channels with minimum energy consumption of the network while not allowing the probability of a false alarm to exceed a certain value. In other words, $P_{f}$ should be kept below a maximum tolerable probability of false alarm (ς), which is defined in next section.

## 4. Proposed Algorithm

**Figure 3:**
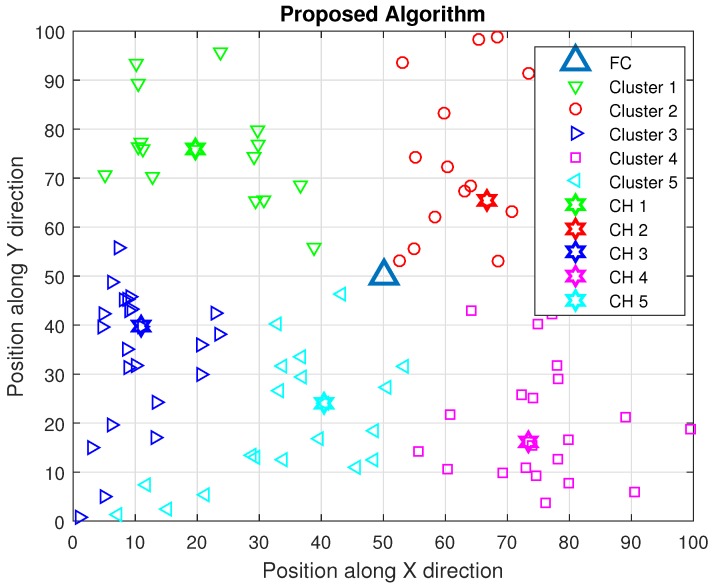
Proposed algorithm.

Our proposed algorithm makes use of four major parameters of the system for the efficient performance, such as distance between CHs and their cluster members, distance between CHs and FC, SNR of the CH and residual energy of the CH. Based on those parameters, the CH with highest SNR and immense residual energy near its cluster members is selected as shown in Figure 3. More specifically, the process of our algorithm is conducted through three steps: the first one is a formation of clusters by FCM, the second one is selection of CH based on location of sensors with respect to FC, location of sensors within the cluster, residual energy of the sensors and SNR of the reporting channel of the sensors, and the third one is local spectrum sensing in each sensor. Once the neighborhood information for all the sensors in the network is defined, the network is split into *M* clusters.

### 4.1. Cluster Formation

All the users in the network are prorated based on a clustering algorithm, such as FCM [27,28]. This method is frequently used in pattern recognition by assigning membership to each data point corresponding to each cluster center, where the summation of membership for all data points should be equal to one. FCM is based on minimization of the following objective function:

(2) $$\min_{\mu_{ij},m_{j}}\left(\upsilon_{q}\right),$$

where

(3) $$\upsilon_{q}=\sum_{i=1}^{N}\sum_{j=1}^{M}\mu_{ij}^{q}∥\theta_{i}-m_{j}{∥}^{2},$$

where *M* is the number of clusters, *q* is the fuzziness exponent greater than 1, $\mu_{ij}$ is the degree of membership of *i*th sensor in cluster *j*, and $m_{j}$ is the center of cluster *j*. The value of $\mu_{ij}$ lies between 0 and 1 for every sensor in the network to each cluster center. This fuzzy partitioning is carried out through iterative optimization of the objective function membership $\mu_{ij}$ and the updated cluster center $m_{j}$ as

(4) $$\mu_{ij}=\frac{1}{\sum_{h=1}^{M}{\left(\frac{∥\theta_{i}-m_{j}∥}{∥\theta_{i}-m_{k}∥}\right)}^{\frac{2}{q-1}}},$$

and

(5) $$m_{j}=\frac{\sum_{i=1}^{N}\mu_{ij}^{q}\cdot \theta_{i}}{\sum_{i=1}^{N}\mu_{ij}^{q}}.$$

The iterative optimization stops when the termination criteria *σ* is met, i.e., $\{\mu_{ij}^{k+1}-\mu_{ij}^{k}\}<\sigma$, whereas *k* is the iteration step. Moreover, the description of cluster formation is shown in Algorithm 1. After cluster formation, the network is split into clusters and the CH selection process is started locally within each cluster.

**Algorithm 1 **Cluster Formation1:*Intitialization : membership values*
$\mu_{i,j}\;\forall \;h=1,2,..M\;\forall \;i=1,2,\ldots N$2:*Cluster Centers Initialized*3:**while**
$\{\mu_{ij}^{k+1}-\mu_{ij}^{k}\}<\sigma$
**do**4: **for**
j=1,2,…M
**do**5:  $m_{j}=\frac{\sum_{i=1}^{N}\mu_{ij}^{q}\cdot \theta_{i}}{\sum_{i=1}^{N}\mu_{ij}^{q}}$6: **end for**7: **for**
i=1,2,..N
**do**8:  **for**
j=1,2,..M
**do**9:   *which is*
$\mu_{ij}^{k}$10:   **if**
$∥\theta_{i}-m_{j}∥>0$
**then**11:    *Calculate*
$\mu_{ij}$
*as*12:    $\mu_{ij}=\frac{1}{\sum_{h=1}^{M}{\left(\frac{∥\theta_{i}-m_{j}∥}{∥\theta_{i}-m_{k}∥}\right)}^{\frac{2}{q-1}}}$13:    *which is*
$\mu_{ij}^{k+1}$14:   **end if**15:  **end for**16: **end for**17:**end while**

### 4.2. Cluster Head Selection

The competition between the candidate sensors to be the CH in a given cluster is based on four parameters given below:

- Location of each candidate sensor within the cluster
- Distance of each candidate sensor with respect to the FC
- SNR of the reporting channel of the CH and FC
- Residual energy of each candidate sensor.

The first parameter led us to select the CH near its clusters members, so that each cluster consumes a less amount of overall energy. The second parameter also plays an important role for selection of CH, in which the intention is that the selected CH should not be very far from FC. We have given priority for the selected CH to be near its cluster members, due to which the distance between CH and FC is increased. Therefore, the third parameter SNR of the reporting channel from CH to FC is considered. Besides distance, location and SNR, the fourth parameter residual energy of the sensor also has a significant role. The residual energy is defined as the sensor’s amount of energy left for the sensing and transmission. Thus, the proposed objective function for the CH selection of the *m*th cluster is given by

(6) $$\Psi_{j}^{\left(m\right)}=\max_{CH}\left(\frac{{℧}_{j}^{\left(m\right)}\gamma_{j}^{\left(m\right)}}{\alpha PL_{j}^{\left(m\right)}+\left(1-\alpha \right)PL_{FC}^{\left(m\right)}}\right),$$

where ${℧}_{j}^{\left(m\right)}$ is the residual energy of the *j*th candidate sensor, $\gamma_{j}^{\left(m\right)}$ is the SNR of reporting channel of *j*th sensor to the FC, $PL_{j}^{\left(m\right)}$ is the average path loss of the channels between the *j*th sensor and its cluster members, $PL_{FC}^{\left(m\right)}$ is the path loss of *j*th sensor and FC, and *α* is a weight given to path losses $PL_{j}^{\left(m\right)}$ and $PL_{FC}^{\left(m\right)}$, i.e., sensor to CH and CH to FC. The $PL_{j}^{\left(m\right)}$ is defined as

(7) $$PL_{j}^{\left(m\right)}=\frac{\sum_{i=1}^{N_{m}}PL_{j,i}^{\left(m\right)}}{N_{m}},$$

where $N_{m}$ is the number of sensors in the *m*th cluster. The $PL_{j,i}^{\left(m\right)}$ is the path loss between *j*th sensor and its cluster member *i*th sensor, which is given by

(8) $$PL_{j,i}^{\left(m\right)}=10\;n\;log_{10}\left(R_{j,i}^{\left(m\right)}\right),$$

where $R_{j,i}^{\left(m\right)}=∥{\theta_{i}}^{\left(m\right)}-\theta_{j}^{\left(m\right)}∥$ is the distance between the *j*th and *i*th sensors with $\theta_{i}^{\left(m\right)}=\left\{x_{i}^{\left(m\right)},y_{i}^{\left(m\right)}\right\},$ the position of *i*th sensor, $\theta_{j}^{\left(m\right)}=\left\{x_{j}^{\left(m\right)},y_{j}^{\left(m\right)}\right\}$ the position of *j*th sensor, and *n* is the path loss exponent. The path loss between CH of *m*th cluster and FC is given as

(9) $$PL_{FC}^{\left(m\right)}=10\;n\;log_{10}\left(R_{FC}^{\left(m\right)}\right),$$

where $R_{FC}^{\left(m\right)}=∥\theta_{j}^{\left(m\right)}-\theta_{\mathrm{FC}}∥$, with $\theta_{\mathrm{FC}}=\left\{x_{fc},y_{fc}\right\}$ the position of FC. The detailed algorithm for CH selection is given in Algorithm 2. Once the CH is selected based on the maximum objective function, the spectrum sensing is carried out.

**Algorithm 2 **Cluster Head Selection1:*Initialization : CH selection for cluster*2:**while**
m=1,2,..,M
**do**3: *Selecting CH f or mth Cluster*4: **for**
$j=1,2,...N_{m,j}$
**do**5:  *Calculate*
${℧}_{j}^{\left(m\right)},\gamma_{j}^{\left(m\right)},PL_{j}^{\left(m\right)}andPL_{FC}^{\left(m\right)}$6:  **if**
$\Psi_{j}^{\left(m\right)}=\max_{CH}\left(\frac{{℧}_{j}^{\left(m\right)}\gamma_{j}^{\left(m\right)}}{\alpha PL_{j}^{\left(m\right)}+\left(1-\alpha \right)PL_{FC}^{\left(m\right)}}\right)$
**then**7:   *mth CH* ← *jth sensor*8:  **else**9:   *Cluster member *←* jth sensor*10:  **end if**11: **end for**12:**end while**

### 4.3. Spectrum Sensing

The eventual goal of the proposed algorithm is to maximize the probability of detection under an imperfect reporting channel with less consumption of energy. Let us denote the received signal by the *i*th sensor of the *m*th cluster for PU’s transmitted signal at *z*th time instant by $y_{i}^{\left(m\right)}\left(z\right)$, which is defined as

(10) $$y_{i}^{\left(m\right)}\left(z\right)∼\left\{\begin{array}{cc} n_{i}^{\left(m\right)}\left(z\right) & H_{0},\\ s\left(z\right)+n_{i}^{\left(m\right)}\left(z\right) & H_{1}, \end{array}\right.$$

where s(z) is the signal transmitted by PU, and $n_{i}^{\left(m\right)}\left(z\right)$ denotes zero-mean additive white Gaussian noise (AWGN) with variance of $\sigma_{i}^{2^{\left(m\right)}}$. The test statistics of the *i*th sensor of *m*th cluster is given by

(11) $$\varpi_{i}^{\left(m\right)}=\sum_{z=0}^{Z-1}{|y_{i}^{\left(m\right)}\left(z\right)|}^{2},\;\;\;\;\;i=1,2,\ldots Z,$$

where *Z* is the total number of samples and $\varpi_{i}^{\left(m\right)}$ is the sum of the squares of *Z* Gaussian independent random variables. It is shown that $\varpi_{i}^{\left(m\right)}$ follows a central chi square $\chi_{cZ}^{2}$ distribution with *Z* degrees of freedom if $H_{0}$ is true; otherwise, it follows non-central $\chi_{ncZ}^{2}$ distribution with *Z* degrees of freedom and non-centrality parameter of *λ*. Thus, we can write it as

(12) $$\varpi_{i}^{\left(m\right)}∼\left\{\begin{array}{cc} \chi_{cZ}^{2} & H_{0},\\ \chi_{ncZ}^{2}\left(\lambda \right) & H_{1}. \end{array}\right.$$

The PDF of $\varpi_{i}^{\left(m\right)}$ can be written as [29]

(13) $$f\left(\varpi_{i}^{\left(m\right)}\right)=\left\{\begin{array}{cc} \frac{{\varpi_{i}^{\left(m\right)}}^{\left(\frac{Z}{2}-1\right)}e^{-\frac{\varpi_{i}^{\left(m\right)}}{2\sigma_{i}^{2^{\left(m\right)}}}}}{2^{Z/2}\Gamma \left(Z/2\right)\sigma_{i}^{Z^{\left(m\right)}}} & H_{0},\\ \frac{1}{2\sigma_{i}^{2^{\left(m\right)}}}{\left(\frac{\varpi_{i}^{\left(m\right)}}{\lambda_{i}^{\left(m\right)}}\right)}^{\frac{Z}{4}-\frac{1}{2}}e^{-\frac{\left(\varpi_{i}^{\left(m\right)}+\lambda_{i}^{\left(m\right)}\right)}{2\sigma_{i}^{2^{\left(m\right)}}}}I_{Z/2-1}\left(\sqrt{\varpi_{i}^{\left(m\right)}\lambda_{i}^{\left(m\right)}}/\sigma_{i}^{2^{\left(m\right)}}\right) & H_{1}, \end{array}\right.$$

where Γ(.) is the gamma function and $I_{v}\left(.\right)$ is the *v*th order modified Bessel function. Thus, both hypotheses can be written as

(14) $$\varpi_{i}^{\left(m\right)}∼\left\{\begin{array}{cc} N(Z\sigma_{i}^{2^{\left(m\right)}},\;2Z\sigma_{i}^{4^{\left(m\right)}}) & H_{0},\\ N(\left(Z+\lambda_{i}^{\left(m\right)}\right)\sigma_{i}^{2^{\left(m\right)}},\;2\left(Z+2\lambda_{i}^{\left(m\right)}\right)\sigma_{i}^{4^{\left(m\right)}}) & H_{1}. \end{array}\right.$$

When *i*th sensor of *m*th cluster forwards the sensing measurement to the *m*th CH under an imperfect reporting channel, the received signal at CH is given by

(15) $$\varpi_{R_{i}}^{\left(m\right)}=\varpi_{i}^{\left(m\right)}h_{R_{i}}^{\left(m\right)}+\omega_{R_{i}}^{\left(m\right)},$$

where $h_{R_{i}}^{\left(m\right)}$ is the channel gain between *i*th sensor of the *m*th cluster, and its CH and $\omega_{R_{i}}^{\left(m\right)}$ is the AWGN of reporting channel with variance $\sigma_{R_{i}}^{2^{\left(m\right)}}$. The mean and variance after normalizing the hypothesis by noise power $\left(\sigma_{R_{i}}^{2^{\left(m\right)}}\right)$ is given as

(16) $$\varpi_{R_{i}}^{\left(m\right)}∼\left\{\begin{array}{cc} N(Z\sigma_{i}^{2^{\left(m\right)}}/\sigma_{R_{i}}^{2^{\left(m\right)}},\;2Z\sigma_{i}^{4^{\left(m\right)}}/\sigma_{R_{i}}^{2^{\left(m\right)}}) & H_{0},\\ N(\left(Z+\lambda_{i}^{\left(m\right)}\right)\sigma_{i}^{2^{\left(m\right)}}\gamma_{i}^{\left(m\right)},\;2\left(Z+2\lambda_{i}^{\left(m\right)}\right)\sigma_{i}^{4^{\left(m\right)}}\gamma_{i}^{\left(m\right)}) & H_{1}, \end{array}\right.$$

where $\gamma_{i}^{\left(m\right)}=\frac{{|h_{R_{i}}^{\left(m\right)}|}^{2}}{\sigma_{R_{i}}^{2^{\left(m\right)}}}$ was defined earlier as the SNR of the reporting channel of *i*th sensor of the *m*th cluster. Sensing measurements of sensors in the cluster are combined at the CH as $\varpi^{\left(m\right)}=\sum_{i=1}^{N_{m}}\varpi_{R_{i}}^{\left(m\right)}$, where $\varpi^{\left(m\right)}$ is the summation of the sensing measurements of all sensors of the *m*th cluster. The mean of all sensors of a cluster is summed up and so is the variance. Assuming each sensor receives the same number of samples, the mean for $H_{0}$ and $H_{1}$ for *m*th cluster is $\sigma_{\mu }^{\left(m\right)}Z$ and $\left(\sigma_{\mu \gamma }^{\left(m\right)}Z+\sigma_{\mu \lambda }^{\left(m\right)}\right)$, respectively, where $\sigma_{\mu }^{\left(m\right)}=\sum_{i=1}^{N_{m}}\sigma_{i}^{2^{\left(m\right)}}/\sigma_{R_{i}}^{2^{\left(m\right)}}$, $\sigma_{\mu \gamma }^{\left(m\right)}=\sum_{i=1}^{N_{m}}\sigma_{i}^{2^{\left(m\right)}}\gamma_{i}^{\left(m\right)}$ and $\sigma_{\mu \lambda }^{\left(m\right)}=\sum_{i=1}^{N_{m}}\sigma_{i}^{2^{\left(m\right)}}\lambda_{i}^{\left(m\right)}\gamma_{i}^{\left(m\right)}$. In addition, the summation of the variance of all sensors for $H_{0}$ and $H_{1}$ is written as $2Z\sigma_{V}^{\left(m\right)}$ and $2(Z\sigma_{V\gamma }^{\left(m\right)}+2\sigma_{V\lambda }^{\left(m\right)})$, where $\sigma_{V}^{\left(m\right)}=\sum_{i=1}^{N_{m}}\sigma_{i}^{4^{\left(m\right)}}/\sigma_{R_{i}}^{2^{\left(m\right)}}$, $\sigma_{V\gamma }^{\left(m\right)}=\sum_{i=1}^{N_{m}}\sigma_{i}^{4^{\left(m\right)}}\gamma_{i}^{\left(m\right)}$ and $\sigma_{V\lambda }^{\left(m\right)}=\sum_{i=1}^{N_{m}}\sigma_{i}^{4^{\left(m\right)}}\lambda_{i}^{\left(m\right)}\gamma_{i}^{\left(m\right)}$. Consequently, the hypothesis at the CH of *m*th cluster is written as

(17) $$\varpi^{\left(m\right)}∼\left\{\begin{array}{cc} N(\sigma_{\mu }^{\left(m\right)}Z,2Z\sigma_{V}^{\left(m\right)}) & H_{0},\\ N(\sigma_{\mu \gamma }^{\left(m\right)}Z+\sigma_{\mu \lambda }^{\left(m\right)},2\left(Z\sigma_{V\gamma }^{\left(m\right)}+2\sigma_{V\lambda }^{\left(m\right)}\right)) & H_{1}. \end{array}\right.$$

Eventually, FC combines sensing measurement received from all CHs, $\varpi =\sum_{m=1}^{M}\varpi^{\left(m\right)}$, where *M* is the total number of clusters. Therefore, the mean and the variance of *ϖ* after combining the sensing measurements received from all CHs become

(18) $$\varpi^{\left(m\right)}∼\left\{\begin{array}{cc} N(Z\sigma_{\mu },2Z\sigma_{V}) & H_{0},\\ N(Z\sigma_{\mu \gamma }+\sigma_{\mu \lambda },2\left(Z\sigma_{V}+2\sigma_{V\lambda }\right)) & H_{1}, \end{array}\right.$$

where $\sigma_{\mu }=\sum_{m=1}^{M}\sigma_{\mu }^{\left(m\right)}$, $\sigma_{\mu \gamma }=\sum_{m=1}^{M}\sigma_{\mu \gamma }^{\left(m\right)}$, $\sigma_{\mu \lambda }=\sum_{m=1}^{M}\sigma_{\mu \lambda }^{\left(m\right)}$, $\sigma_{V}=\sum_{m=1}^{M}\sigma_{V}^{\left(m\right)}$, $\sigma_{V\gamma }=\sum_{m=1}^{M}\sigma_{V\gamma }^{\left(m\right)}$ and $\sigma_{V\lambda }=\sum_{m=1}^{M}\sigma_{V\lambda }^{\left(m\right)}$. Using the Neyman–Pearson Lemma, the optimum soft information-combining strategy for the proposed algorithm with threshold *φ* is given by

(19) $$\frac{P(\varpi |H_{1})}{P(\varpi |H_{0})}≷\varphi .$$

For determining the maximum tolerable false alarm probability (ς), which is defined as $\int_{\varphi }^{\infty }P\left(H_{0}\right)$,

(20) $$\varsigma =\int_{\varphi }^{\infty }\frac{1}{\sqrt{8\pi Z\sigma^{\left(V\right)}}}e^{\frac{-1}{2Z\sigma^{\left(V\right)}}{\left(\varpi -Z\sigma^{\left(\mu \right)}\right)}^{2}}d\varpi ,$$

which can be written as

(21) $$\varphi =Q^{-1}\left(P_{f}\right)\sqrt{2Z\sigma^{\left(V\right)}}+Z\sigma^{\left(\mu \right)}.$$

The probability of detection which is $\int_{\varphi }^{\infty }P\left(H_{1}\right)$ is computed as

(22) $$P_{d}=\int_{\varphi }^{\infty }\frac{1}{\sqrt{4\pi (2\left(Z\sigma^{\left(V\gamma \right)}+2\sigma^{\left(V\lambda \right)}\right))}}e^{\frac{-{\left(\varpi -\sigma^{\left(\mu \gamma \right)}Z-\sigma^{\left(\mu \lambda \right)}\right)}^{2}}{2(Z\sigma^{\left(V\gamma \right)}+2\sigma^{\left(V\lambda \right)})}}d\varpi .$$

The above equation after substituting (21) becomes

(23) $$P_{d}=Q\left(\frac{Q^{-1}\left(P_{f}\right)\sqrt{2Z\sigma^{\left(V\right)}}+Z\sigma^{\left(\mu \right)}-\left(Z\sigma^{\left(\mu \gamma \right)}+\sigma^{\left(\mu \lambda \right)}\right)}{\sqrt{2(Z\sigma^{\left(V\gamma \right)}+2\sigma^{\left(V\lambda \right)})}}\right).$$

The above equation is probability of detection computed from the sensing measurements transmitted by CHs to FC.

### 4.4. Energy Efficiency Analysis

The goal of this subsection is to show that the proposed algorithm achieves the highest performance of the network with a small consumption of energy. The performance of the network can be found by the energy efficiency metric, which is defined as the ratio of average throughput of the network and energy consumed by the network. Typically, the energy consumed by a single sensor is due to sensing power and transmission power for reporting the sensed data. The amounts of energy required to sense and transmit the sensed data to CH over a transmission distance of *R* is given by [30].

(24) $$E_{m,i}=T_{s}P_{s}+R_{j,i}^{\left(m\right)}P_{t,i}^{\left(m\right)},$$

where $R_{j,i}^{\left(m\right)}$ was defined earlier, $T_{s}$, $P_{s}$ and $P_{t,i}^{\left(m\right)}$ denote sensing time, power consumption due to sensing and power consumption of the *i*th sensor of the *m*th cluster due to transmission. Each sensor forwards sensing measurement to its CH. Hence, the energy consumed by a *m*th cluster is given by

(25) $$E_{m}=N_{m}T_{s}P_{s}+\sum_{i=1}^{N_{m}}R_{j,i}^{\left(m\right)}P_{t,i}^{\left(m\right)},$$

where $N_{m}$ is the number of sensors in the *m*th cluster. The CHs after combining the sensing measurements of all of their sensors forward the combined sensing measurement to FC. Thus, the total energy consumed by a network is given by

(26) $$E=NT_{s}P_{s}+\sum_{m=1}^{M}\sum_{i=1}^{N_{m}}\left(R_{j,i}^{\left(m\right)}+R_{FC}^{\left(m\right)}\right)P_{t,i}^{\left(m\right)}+\left(1-P_{0}P_{f}-P_{1}P_{d}\right)P_{t,i}^{\left(m\right)}T_{t},$$

where $R_{FC}^{\left(m\right)}$ was defined earlier, *M* is the total number of clusters, $P_{0}$ is the probability that the spectrum is unused, $P_{1}$ is the probability that the spectrum is used and $T_{t}=\left(T-T_{s}-\left(N/M\right)T_{r}-MT_{r}\right)$ with *N* the total number of sensors in the network. The throughput is defined as the amount of successful delivery of data of all sensors, which is given by

(27) $$\beta =P_{0}\left(1-P_{f}\right)B\left(T-T_{s}-N_{m}T_{r}-MT_{r}\right)+P_{1}\left(1-P_{d}\right)BT_{t},$$

where *B* is the data rate. The energy efficiency, which is average throughput of the network over energy consumed by the network is given by [30]

(28) $$\varepsilon =\frac{\beta }{E}.$$

The above equation is used to find the efficiency of the proposed algorithm.

## 5. Simulation Results

**Figure 4:**
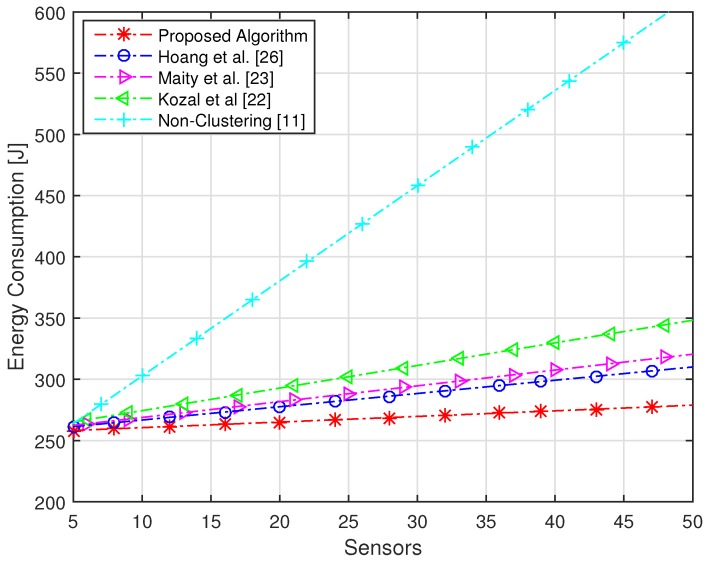
Energy consumption with an increasing number of sensors.

**Figure 5:**
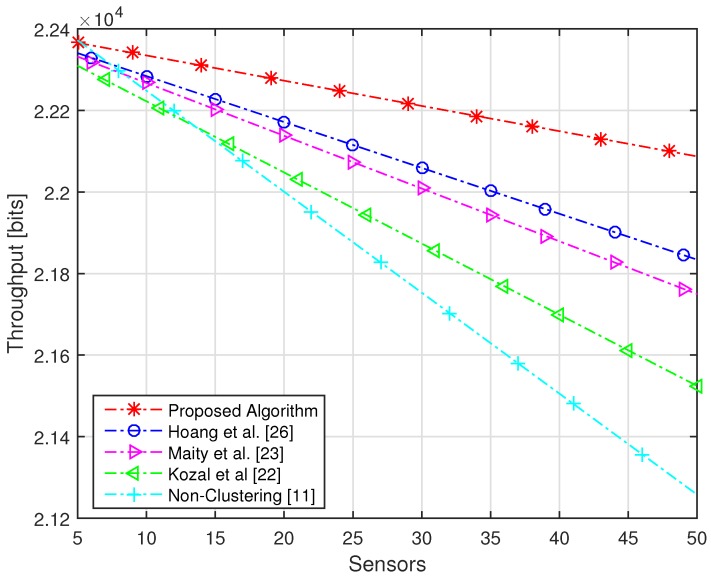
Throughput with an increasing number of sensors.

**Figure 6:**
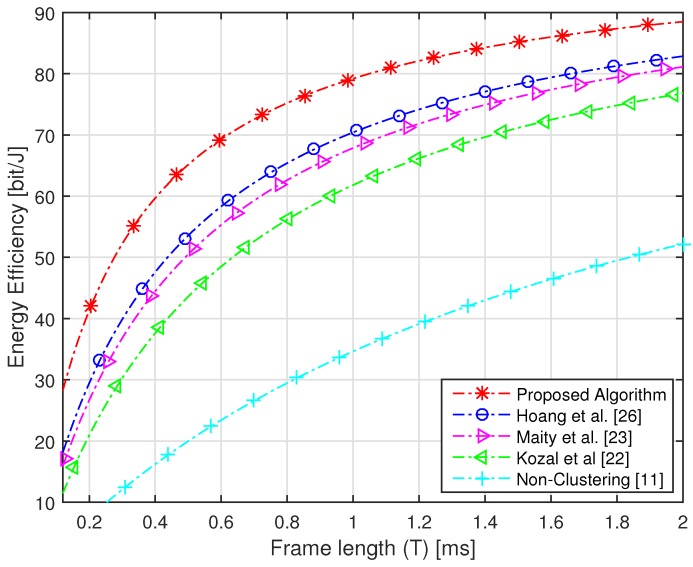
Energy efficiency with an increasing time frame.

**Figure 7:**
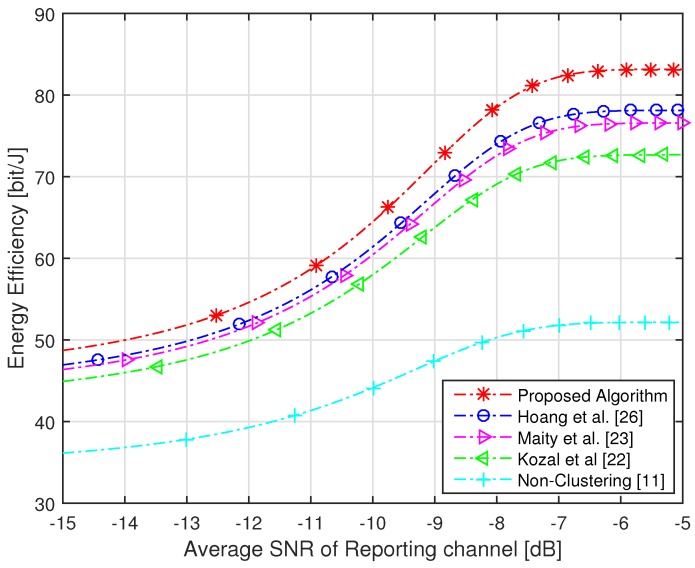
Energy efficiency with an increasing signal-to-noise ratio (SNR) of the reporting channel.

The target of the proposed algorithm is to acquire the highest sensing performance under an imperfect reporting channel with maximum energy efficiency. The sensing performance is dependent on $P_{d}$ and $P_{f}$, while energy efficiency is defined by energy consumption and throughput. The performance of the proposed algorithm is verified by comparing the performance with non-clustering and conventional clustering schemes. The network consists of a maximum of 100 numbers of sensors, which are divided into five numbers of clusters with each cluster having random numbers of sensors assigned based on FCM. We have compared our algorithm with [11,22,23,26]. In [11], sensors of the whole network forward their sensing information to the FC, due to which the highest amount of energy is consumed. In [22], a modified version of cluster making method LEACH is proposed, in which sensors share their information with neighbor nodes. In [23], the network is divided into clusters using FCM and every sensor forwards its sensing energy to FC. In [26], authors make the cluster using FCM and select the CH based only on residual energy. Due to the small distance between CHs and their cluster members in the proposed algorithm, a very small amount of energy is consumed and highest throughput is achieved as compared to conventional clustering schemes. The energy consumption of the proposed algorithm is compared with non-clustering and conventional clustering schemes with an increasing number of sensors in Figure 4. It is clear from the figure that the proposed algorithm consumes a less amount of energy as compared to conventional clustering schemes. It is shown that the non-clustering cooperative spectrum sensing consumes the highest energy as compared to clustering schemes. Furthermore, we can see from the figure that 50 numbers of sensors in the whole network [22,23,26] consume more than 300 J of energy, while with the same number of sensors, the proposed algorithm consumes energy of approximately 260 J. The throughput of the proposed algorithm and conventional schemes is shown in Figure 5, which clearly illustrates that, using the proposed algorithm, the highest throughput of the network is achieved as compared to conventional schemes. The energy efficiency of the network is increased with increasing frame time, as the network has a huge amount of time for transmission of data. Due to small energy consumption and large throughput, the proposed algorithm has the highest efficiency as compared to conventional clustering schemes with increasing time frame. Figure 6 has compared the energy efficiency of the proposed algorithm and conventional clustering schemes with increasing time frame. It is worth noting that maximum energy efficiency is achieved by using the proposed algorithm as compared to conventional schemes. With total time frame of 2 ms, conventional schemes achieve energy efficiency of nearly 80 bit/J, while with same period of time, the proposed algorithm obtains the energy efficiency of approximately 90 bit/J. We have compared the efficiency of the network with increasing average SNR of the reporting channel. The SNR of channels play an important role; therefore, we used this parameter for the selection of CH. We have achieved highest energy efficiency using the proposed algorithm with increasing SNR of the reporting channel, as shown in Figure 7. It is clear from the figure that, with −5 dB of SNR, conventional schemes acquire energy efficiency of less than 80 bit/J, while with the same average SNR of the reporting channels, the proposed algorithm achieves energy efficiency of more than 80 bit/J. Each sensor consumes tremendous energy if it has a large distance from its CH.

**Figure 8:**
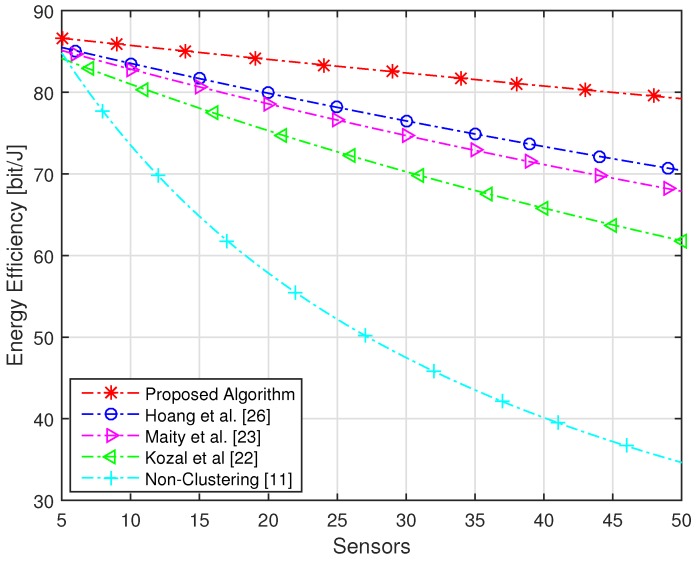
Energy efficiency of the whole network with an increasing number of sensors.

In our proposed algorithm, this distance is short, which led us to achieve the highest energy efficiency. The energy efficiency of the proposed algorithm with an increasing number of sensors is compared with conventional schemes in Figure 8. It is clear from the Figure that, using the proposed algorithm with 50 numbers of sensors, we still have energy efficiency of 80 bit/J, while using conventional schemes with the same number of sensors, the achieved energy efficiency is equal to or below 70 bit/J.

## 6. Conclusions

In this paper, a clustering technique is proposed that uses the fuzzy c-means clustering method for the formation of clusters and cluster head is selected based on four parameters: sensor’s location within cluster, location with respect to FC, its SNR, and its residual energy. The goal of our algorithm is to get the highest performance of the network under imperfect channels with a smaller consumption of energy. The proposed algorithm selects the CH near its cluster members with high SNR and residual energy, which saves a large amount of energy. We have shown that our proposed algorithm performs better than conventional clustering techniques in terms of detection performance and consumes less energy as compared to them.

## Abbreviations

FCM	Fuzzy c-means clustering
CH	Cluster head
FC	Fusion center
SNR	Signal-to-noise ratio
PU	Primary user
RSSI	Received signal strength indicator
LEACH	Low energy adaptive clustering hierarchy
AWGN	Additive white Gaussian noise
